# Efficient production of immunologically active *Shigella* invasion plasmid antigens IpaB and IpaH using a cell-free expression system

**DOI:** 10.1007/s00253-021-11701-4

**Published:** 2021-12-21

**Authors:** Neeraj Kapoor, Esther Ndungo, Lucy Pill, Girmay Desalegn, Aym Berges, Edwin V. Oaks, Jeff Fairman, Marcela F. Pasetti

**Affiliations:** 1 Vaxcyte, Inc., 353 Hatch Dr, Foster City, CA 94404 USA; 2 Center for Vaccine Development and Global Health, University of Maryland School of Medicine, 685 W. Baltimore Street, Baltimore, MD 21201 USA; 3 Patuxent Research and Consulting Group, Gambrills, MD USA

**Keywords:** *Shigella*, Cell-free protein synthesis, Vaccine, Invasion plasmid antigens

## Abstract

**Abstract:**

*Shigella* spp*.* invade the colonic epithelium and cause bacillary dysentery in humans. Individuals living in areas that lack access to clean water and sanitation are the most affected. Even though infection can be treated with antibiotics, *Shigella* antimicrobial drug resistance complicates clinical management. Despite decades of effort, there are no licensed vaccines to prevent shigellosis. The highly conserved invasion plasmid antigens (Ipa), which are components of the *Shigella* type III secretion system, participate in bacterial epithelial cell invasion and have been pursued as vaccine targets. However, expression and purification of these proteins in conventional cell-based systems have been challenging due to solubility issues and extremely low recovery yields. These difficulties have impeded manufacturing and clinical advancement. In this study, we describe a new method to express Ipa proteins using the Xpress^+TM^ cell-free protein synthesis (CFPS) platform. Both IpaB and the C-terminal domain of IpaH1.4 (IpaH-CTD) were efficiently produced with this technology at yields > 200 mg/L. Furthermore, the expression was linearly scaled in a bioreactor under controlled conditions, and proteins were successfully purified using multimode column chromatography to > 95% purity as determined by SDS-PAGE. Biophysical characterization of the cell-free synthetized IpaB and IpaH-CTD using SEC-MALS analysis showed well-defined oligomeric states of the proteins in solution. Functional analysis revealed similar immunoreactivity as compared to antigens purified from *E. coli.* These results demonstrate the efficiency of CFPS for *Shigella* protein production; the practicality and scalability of this method will facilitate production of antigens for *Shigella* vaccine development and immunological analysis.

***Key points*:**

• *First report of Shigella IpaB and IpaH produced at high purity and yield using CFPS*

• *CFPS-IpaB and IpaH perform similarly to E. coli–produced proteins in immunoassays*

• *CFPS-IpaB and IpaH react with Shigella-specific human antibodies and are immunogenic in mice.*

**Graphical abstract:**

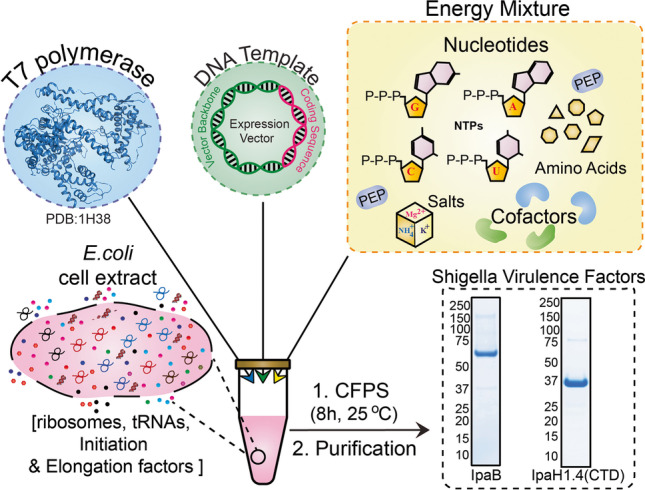

## Introduction

**Fig. 1:**
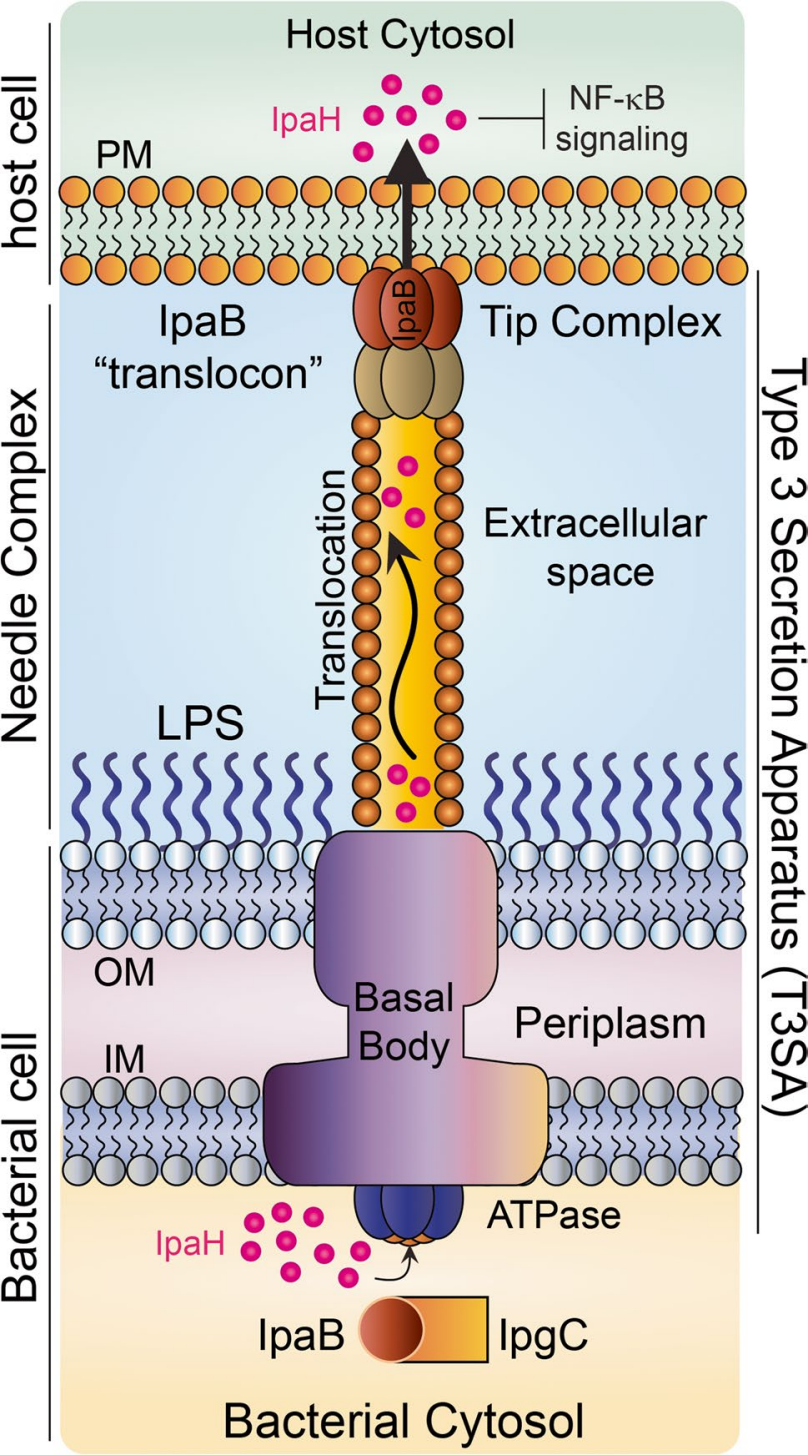
Schematic representation of the *Shigella* type III secretion apparatus (T3SA) and role of IpaB and IpaH proteins. IpaB binds to its cognate chaperone IpgC in the bacterial cytosol. *Shigella* T3SA extends from the bacterial inner and outer membrane and across the periplasm to form a pore into the host cell plasma membrane. At the tip of this injection needle, IpaB assembles into a pore-forming oligomeric translocon that facilitates delivery of virulence factors. The IpaH family of proteins are effectors secreted through the T3SA and are involved in modulating host immune responses. IpaH suppresses NF-κB activation, which downregulates inflammatory responses

*Shigella* spp. are a leading cause of moderate to severe diarrhea in young children living in low- and middle-income countries and the second cause of diarrheal mortality among all ages (Khalil et al. 2018; Kotloff et al. 2019; Livio et al. 2014). *Shigella* invasion, replication, and spread within the colonic epithelium result in acute dysentery (bloody, mucoidal diarrhea). Although antibiotic treatment can limit the disease, the increased prevalence of antibiotic resistance among *Shigella* isolates demands improved preventive measures including better hygiene, clean water, and pathogen-specific immunity. Efforts to produce *Shigella* vaccines have been ongoing for decades, and yet a safe and effective vaccine has not materialized. A hindrance to this endeavor has been the incomplete understanding of bacterial pathogenesis, the underlying mechanisms of host defenses, and antigen specificity required for protective immunity. Several vaccine candidates are in various clinical stages of development (Walker et al. 2021), most of which rely on generating immunity against the *Shigella* O-antigen. The O-antigen vaccine concept stems from epidemiological evidence of antibody-associated O-serotype-specific protection (Cohen et al. 1991; Cohen et al. 2019; Robin et al. 1997). A logistic drawback of this approach is the need for multiple O-antigen vaccine components to prevent disease caused by different circulating serotypes. In addition, bacterial polysaccharides require bystander T helper–inducing molecules to generate strong and long-lasting adaptive immunity (Avci et al. 2019; Rappuoli 2018). These requirements increase complexity of manufacturing and cost. Other major vaccine targets are the conserved invasion plasmid antigen (Ipa) proteins (Fig. 1) (Heine et al. 2013; Martinez-Becerra et al. 2013a; Martinez-Becerra et al. 2012; Riddle et al. 2011; Turbyfill et al. 2018). The Ipa proteins are part of the *Shigella* type III secretion system (T3SS), a molecular machine that injects bacterial virulence effectors into host cells (Fig. 1) (Bajunaid et al. 2020; Schnupf and Sansonetti 2019; Schroeder and Hilbi 2008), a critical initial step in *Shigella* invasion of colonic epithelial cells.

IpaB is a 62 kDa hydrophobic protein highly conserved among *Shigella* species (homology > 98%) that functions as a T3SS translocator and effector. It is synthesized in the bacterial cytoplasm, complexed with its cognate chaperone IpgC, recruited after IpaD to the needle tip, and eventually secreted as a complex with IpaC that forms a pore in the host cell membrane (Fig. 1). IpaB interaction with the host cell membrane is required for *Shigella* invasion. IpaB is also critical in the process by which *Shigella* escape from the phagosome and enter the host cell cytoplasm and is responsible for apoptosis of phagocytic cells (Mattock and Blocker 2017; Schnupf and Sansonetti 2019; Schroeder and Hilbi 2008). Antibodies to IpaB are prominent in infected individuals living in endemic areas and in subjects orally immunized with live attenuated strains (Ndungo et al. 2018; Oaks et al. 1986; Oberhelman et al. 1991; Van de Verg et al. 1992). IpaB-specific IgG (IgG1) titers have been associated with clinical protection against shigellosis in experimentally infected human volunteers (Shimanovich et al. 2017). Several *Shigella* vaccine candidates based on IpaB have been evaluated in animal models (Chitradevi et al. 2015; Chitradevi et al. 2016; Heine et al. 2014; Heine et al. 2013; Martinez-Becerra et al. 2012; Martinez-Becerra et al. 2013b). Despite the encouraging proof of principle efficacy in pre-clinical studies, IpaB has never been evaluated in humans as a purified vaccine candidate. The Ipa proteins have been traditionally difficult to produce at large scale due to solubility and yield issues. IpaB has been purified post expression in *E. coli* with only moderate yields (1–2 mg/L culture) (Picking et al. 1996), and the low solubility of the protein requires co-expression of its cognate chaperone IpgC, which is a limiting factor in cell-based expression systems (Birket et al. 2007; Dickenson et al. 2013).

The IpaH family of proteins, which are also T3SS effector proteins, are widely conserved among *Shigella* and/or other closely related bacteria (Ashida and Sasakawa 2015). IpaH proteins contain an N-terminal leucine-rich repeat and a C-terminal region with E3 ubiquitin ligase activity (Ashida et al. 2007). While the N-terminal domain varies among IpaH proteins (encoded by either the chromosome or virulence plasmid), the C-terminal domain (IpaH-CTD) is conserved between isoforms and *Shigella* species (Ashida and Sasakawa 2015). IpaH-CTD has been shown to possess E3 ubiquitin ligase activity which promotes bacterial survival by triggering macrophage killing and dampening the host immune responses through NF-κB inhibition (Ashida and Sasakawa 2015; Rohde et al. 2007; Singer et al. 2008). *ipa*H genes are present in the genomes of all *Shigella* species and thus have long been used as targets in molecular diagnostics of *Shigella* and/or the closely related enteroinvasive *E. coli* (EIEC) in fecal samples (Lindsay et al. 2013; Liu et al. 2016; Sahl et al. 2015; Venkatesan et al. 1989; Vu et al. 2004). Nonetheless, evidence of immunogenicity of IpaH had been limited until recently when our group demonstrated human serum antibody reactivity to IpaH in a novel microarray system (Ndungo et al. 2018). Functional and structural studies on IpaH have been performed with purified IpaH or portions of IpaH (Ye et al. 2020), and as with IpaB, these studies report production of small quantities of protein using traditional research-scale recombinant methods.

**Fig. 2:**
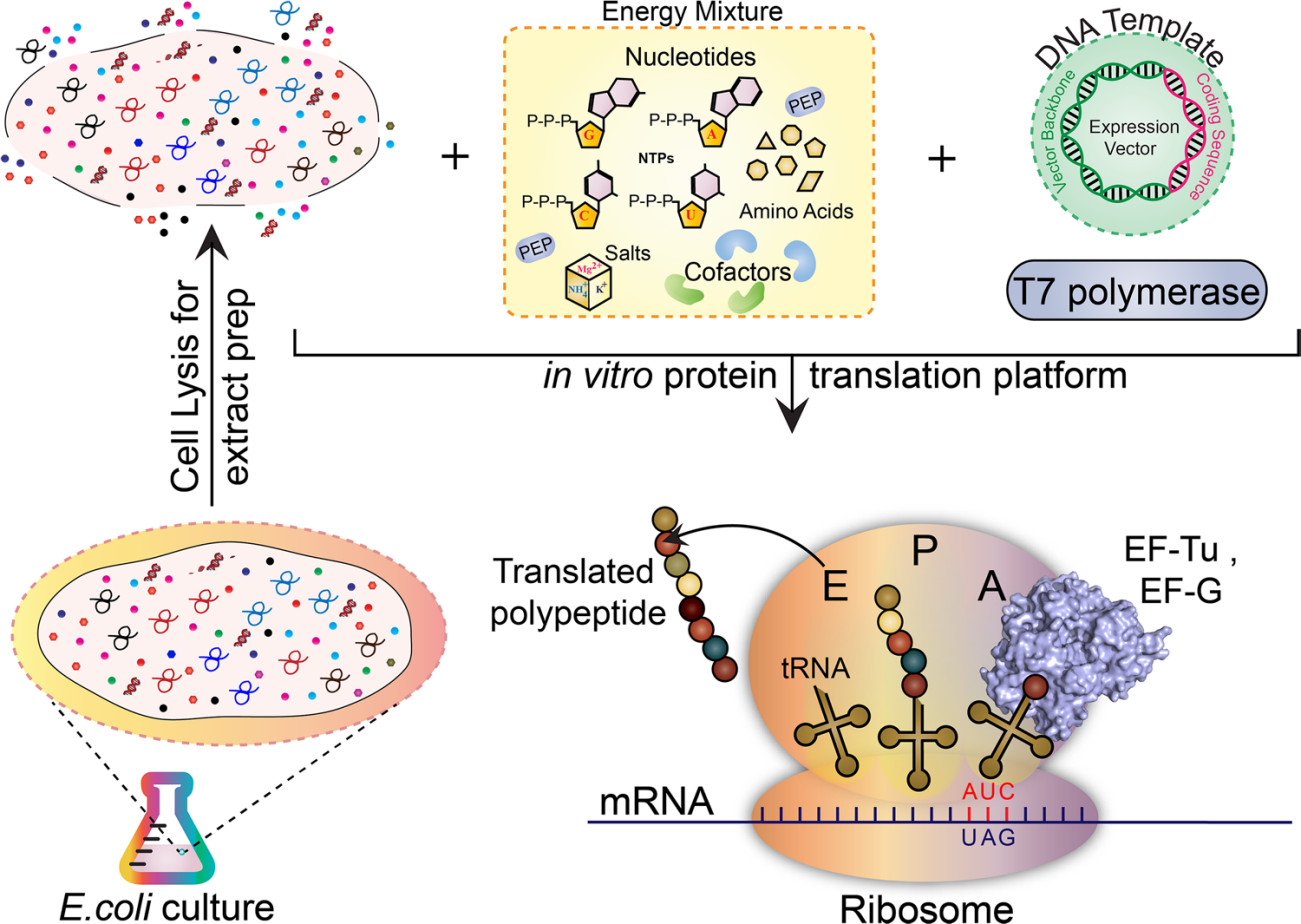
Schematic representation of the Xpress^+TM^ CFPS technology. For CFPS, cellular extract from *E. coli* is harvested and added to a chemical mixture containing components (NTPs, amino acids, Na^+^/K^+^ salts and cofactors) necessary for energy generation and transcription (through simultaneous addition of plasmid DNA encoding the target gene and T7 polymerase). Thereafter, the transcribed mRNA is readily utilized by the ribosomal translational machinery in the cellular extract to express the target protein in 8-10h.

A *Shigella* vaccine based on shared proteins is appealing for its simplicity and, unlike the O-antigen-based counterparts, can broaden effectiveness against multiple *Shigella* serotypes. New technologies for simple and efficient production of protein vaccine candidates are necessary to eventually achieve a scalable product that can be manufactured consistently and clinically evaluated. Here, we describe the adaptation and optimization of the Xpress^+TM^ cell-free protein synthesis (CFPS) platform to express and purify high yields of full-length IpaB and IpaH-CTD (i.e., C-terminal domain of IpaH1.4). The Xpress^+TM^ CFPS (Fig. 2) is a simple method for protein expression that employs DNA encoding a protein of interest and a synthesis reaction (cellular extract) mix, which contains amino acids and all essential biochemical components for gene transcription, translation, and protein production (including source of energy). Because it is independent of cell (e.g., *E. coli*) viability, the CFPS enables expression of proteins that are toxic for the cell substrate (Kapoor et al. 2018; Xu et al. 2015; Zawada et al. 2011). The absence of a cellular membrane creates an open system whereby components of the synthesis reaction can be manipulated to enhance transcription, translation, and folding. Unlike cell-based systems, only the gene of interest is transcribed and translated, which results in rapid and high yield protein production: typically, g/L yields in 8–10 h. The process is scalable up to > 1000 L (Zawada et al. 2011), and protein can be manufactured under GMP at industry-level quantities within a few days. Using CFPS, both IpaB and IpaH-CTD were expressed at yields > 200 mg/L while the biophysical and antigenic characterization of the purified proteins indicated well-defined solution state structures harboring important conformational and immunologically relevant epitopes. The CFPS can therefore produce protein quantities compatible with industry manufacturing standards (Zawada et al. 2011) and thus facilitate the synthesis of these well-characterized antigens in a reliable and cost-effective manner for their evaluation as *Shigella* vaccine antigens or immune/diagnostic antigens.

## Materials and methods

### Cloning, expression, and purification of IpgC, IpaB, and IpaH-CTD

The genes for expression of IpgC (aa 1-155, GenBank # AAP78992.1), IpaB (aa 1-580, GenBank # SVH88885.1), and the C-terminal domain of IpaH1.4 (IpaH-CTD, aa 265-575, GenBank # AAP79042.1) from *S. flexneri* 2a were synthesized at ATUM (Menlo Park, CA) and subcloned with an N-terminal methionine into a proprietary vector using NdeI and SalI as restriction sites. To facilitate protein capture and purification, IpaB was expressed with a C-terminal his_6_-tag, while full-length IpgC was expressed with a cleavable N-terminal Twin Strep tag. To generate an untagged version of IpgC, the expression constructs harbored a TEV protease cleavage site C-terminal to the affinity tag. For exogenous addition of untagged IpgC during synthesis, IpaB was expressed at large scale using a DASbox mini bioreactor system (Eppendorf, Enfield, CT) with 3 μg/ml of plasmid DNA added to the reaction. Expression was performed at 25 °C for 10 h with constant stirring at 650 rpm while maintaining the pH at 7.2 and sparging a blend of air and oxygen through the reaction mixture to maintain the dissolved oxygen at 30%. After 10 h, the reactions were harvested and spun down at 15,000g at 4 °C for 30 min followed by filtration using a 0.45-μm pore size membrane. For purification, the clarified filtrate with IpgC was loaded onto a 5ml StrepTactin XT column (IBA life sciences, Germany) equilibrated with Buffer A1 (50 mM Tris, 150 mM NaCl) followed by 30 column volumes (CVs) wash with Buffer A1 to bring the A_280_ nm absorbance back to baseline. The bound protein was eluted using Buffer A1 supplemented with 50 mM biotin in a single step. The elution fractions were pooled and incubated with excess in-house purified his_6_-tagged TEV protease while dialyzing against Buffer A1. The next day, the dialyzed cleavage reaction was loaded back onto a pre-equilibrated HisTrap excel 5 ml column (Cytiva, Sweden) followed by a StrepTactin XT column (IBA life sciences, Germany) to separate and purify the untagged IpgC away from the his_6_-tagged protease and the cleaved Twin Strep tag. Next, IpaB was expressed similarly in the CFPS in the presence of exogenously added IpgC (0.3 mg/ml). For purification, post-expression clarified filtrate was loaded onto a 5 ml HisTrap excel column pre-equilibrated with Buffer A2 (50 mM Tris, 150 mM M NaCl, 10 mM Imidazole, 0.1% LDAO), which in turn also helped remove co-eluting IpgC from IpaB. Finally, the bound protein was eluted using a 50% step gradient of Buffer A2 with 500 mM imidazole. Post-capture, the elution fractions for IpaB were combined, concentrated using 30 kDa cutoff Amicon® Ultra-15 centrifugal filters (Millipore Sigma, USA), and loaded onto a gel filtration Superdex200 26/60 column pre-equilibrated with Buffer S1 (50 mM Tris, 150 mM M NaCl, 0.1% LDAO). Fractions with the highest purity (> 95% as assessed by SimplyBlue^TM^ SafeStain (ThermoFisher, USA) staining post SDS-PAGE analysis were combined, aliquoted, and stored at – 80 ^°^C for further use. The expression and purification for IpaH-CTD were performed similarly and post elution from HisTrap excel 5 ml column using Buffer A3 (50 mM Tris, 150 mM M NaCl, 10 mM imidazole); the fractions were concentrated and loaded onto a Superdex75 26/60 column pre-equilibrated with Buffer S2 (50 mM Tris, 150 mM M NaCl). The elution fractions were analyzed by SDS-PAGE analysis followed by SimplyBlue^TM^ SafeStain safe blue staining. Fractions with the highest purity (> 95%) were combined, concentrated using 30 kDa cutoff Amicon® Ultra-15 centrifugal filters (Millipore Sigma, USA), and aliquoted for storage at – 80 °C. The concentration was measured by A_280_ nm while subtracting the background absorbance for the buffer alone.

### ^14^C-leucine incorporation assay for estimate of protein expression

The amount of total and soluble protein expressed using Xpress CFPS^+TM^ platform was determined through ^14^C-leucine incorporation as described previously (Kapoor et al. 2018). ^14^C-leucine of 2 mM (GE Life Sciences, Piscataway, NJ) was added into the CFPS reaction mix and incorporated into the translating polypeptide at 25 °C. Post expression, reactions were harvested, and 4 μl of either the complete CFPS reaction or the clarified supernatant (obtained after centrifuging the reaction at 4500 rpm for 15 min at 25 °C) was blotted onto an anion exchange filter membrane. The membrane was extensively washed to remove unbound material and heat dried for 30 min. Finally, the filter membrane was evenly coated with scintillation fluid, air-dried, and the counts recorded to estimate the total and soluble yield of the expressed proteins. Using these values, final titers were estimated using the formula:

$$Titer= Recorded\ Counts\ \left( Total\ or\ Soluble\ post\ wash\right)\ast \frac{\left(\frac{2\ mM\ast protein\ molecular\ mass}{\# Leucines}\right)}{Total\ Recorded\ Counts}$$

### Western blot analysis

Post CFPS, reactions were spun down at 4500 rpm to harvest the supernatant and pellet fractions. Thereafter, 10 μl of supernatant and corresponding pellet were separately incubated with 4× LDS loading buffer heated at 75 °C for 10 min followed by SDS-PAGE analysis using Nu-PAGE Bis-Tris 4–12% precast gels (Thermo Fisher Scientific, Waltham, MA). The size-separated proteins in the gel were then transferred onto a PVDF membrane using an iBlot apparatus (Thermo Fisher Scientific, Waltham, MA) following the manufacturer’s protocol. After transfer, the membrane was blocked with 50 mM Tris pH 8.0, 50 mM NaCl, and 5% BSA for 30 min followed by incubation with 1:10,000 diluted penta-his HRP monoclonal antibody (Qiagen, Hilden, Germany) for 30 min at RT with constant shaking. After incubation, the membrane was washed 3× with 50 mM Tris pH 8.0, 50 mM NaCl, and 5% BSA at RT for 30 min each. Finally, the membrane was dried and probed with super signal chemiluminescent pico substrate (ThermoFisher Cat#34577) before imaging using Syngene G-Box F3 image scanner (Syngene, Frederick, MD).

### Multi-angle light scattering (MALS) analysis

The SEC MALS-UV-RI setup consists of an Agilent HPLC 1100 degasser, temperature-controlled auto-sampler (4 °C), column compartment (25 °C),and UV-Vis diode array detector (Agilent, Santa Clara, CA) in line with a DAWN-HELEOS multi-angle laser light scattering detector and Optilab T-rEX differential refractive interferometer (Wyatt Technology, Santa Barbara, CA). The system was coupled to a Superdex200 10/30 Increase column for IpaB and Superdex75 10/30 GL for IpaH-CTD. A mobile phase consisting of 0.2 μm filtered 50 mM Tris pH 8, 150 mM NaCl, 0.1% (v/v) LDAO for IpaB or just 50 mM Tris pH 8, and 150 mM NaCl for IpaH-CTD was used at a 0.5 mL/min flow rate. Approximately 50–100-μg sample was injected for analysis. Agilent Open Lab software was used to control the HPLC, and Wyatt Astra 7 software (Wyatt Technology Corp., Santa Barbara, CA) was used for data collection and molecular weight analysis.

### Clinical samples and analysis of IpaB and IpaH-CTD antibody reactivity

Serum samples were obtained from two previous clinical studies performed on healthy community volunteers at the Center for Vaccine Development (University of Maryland, Baltimore) under IRB-approved protocols. The studies were:

(i) *S.flexneri 2a* human challenge: Serum samples were obtained at days -1 (prior to challenge) and 28 (post challenge) from 14 volunteers who were fed 1 × 10^3^ CFU of the wild-type strain *S.flexneri 2a* strain 2457T (ATCC # 700930) as described previously (Kotloff et al. 1995). Specimens were selected from volunteers who remained healthy, as well as from those who experienced mild, moderate, and severe disease, as previously described (Shimanovich et al. 2017).
(ii) CVD 1204 vaccination: Serum samples were collected from 5 subjects orally immunized with a single dose of 1 × 10^9^ CFU of live attenuated *S.flexneri 2a* vaccine strain CVD 1204 (produced at University of Maryland, Baltimore) (Noriega et al. 1996), which harbors deletion mutations in genes encoding enzymes in the guanine nucleotide synthesis pathway (Δ*guaBA*), in a phase I clinical study (Kotloff et al. 2004). Serum samples collected at day -1 (prior to vaccination) and day 28 (post vaccination) were tested.

### Mouse immunizations

Adult (6–8 weeks old) female BALB/c mice (*n* = 10–20 per group, Charles River Lab) were immunized intramuscularly on days 0, 14, and 28 with 10 μg CFPS-purified IpaB or IpaH-CTD adsorbed to Adju-Phos® (4.8% v/v) in a 100-μL volume (50μL per leg). Blood was collected at day -1 (one day prior to vaccination), and days 13, 27, 42, and 55 (post vaccination) for serum antibody measurement. Control groups received PBS or AdjuPhos®. Studies were approved by the University of Maryland Institutional Animal Care and Use Committee.

### Antibody measurements

Antigen-specific serum IgG titers specific for IpaB and IpaH-CTD were measured by ELISA as previously described (Martinez-Becerra et al. 2012; Shimanovich et al. 2017). Briefly, Immulon 2HB plates (Thermo Scientific, Waltham, MA) were coated with IpaB or IpaH-CTD at 0.1 μg/mL in PBS. Plates were incubated for 3 h at 37 °C and blocked at 4 °C overnight in PBS containing 10% non-fat dry milk (NFDM). Sera serially diluted in PBS containing 10% NFDM and 0.05% Tween-20 (PBS-T) were added, and the plates incubated at 37 °C for 1 h. Plates were incubated with HRP-labeled goat IgG specific for human or mouse IgG (Jackson Immuno Research, West Grove, PA) for another 1 h at 37 °C. Plates were washed 6 times with PBS-T following every incubation step. Tetramethylbenzidine (TMB; KPL, Gaithersburg, MD) was added as substrate for 15 min in the dark with shaking, and the reaction was stopped by adding 100 μl 1M phosphoric acid (Millipore Sigma, Burlington, MA). Plates were read using an Multiskan Accent^TM^ Microplate Reader (Thermo Scientific, Waltham, MA). Endpoint titers were calculated by interpolation of absorbance values of samples in the regression curve of a positive control and were reported as ELISA units/mL corresponding to the inverse serum dilution resulting in an A_450_ of 0.2 above background.

### Statistical analysis

Antibody titers were analyzed by paired *t*-test. Differences were considered statistically significant at *p* < 0.05. All statistical analysis was conducted using GraphPad Prism 9 (GraphPad Software, La Jolla, CA).

## Results

### Exogenous addition of IpgC to CFPS enhances expression, solubility, and recovery yields of IpaB

**Fig. 3:**
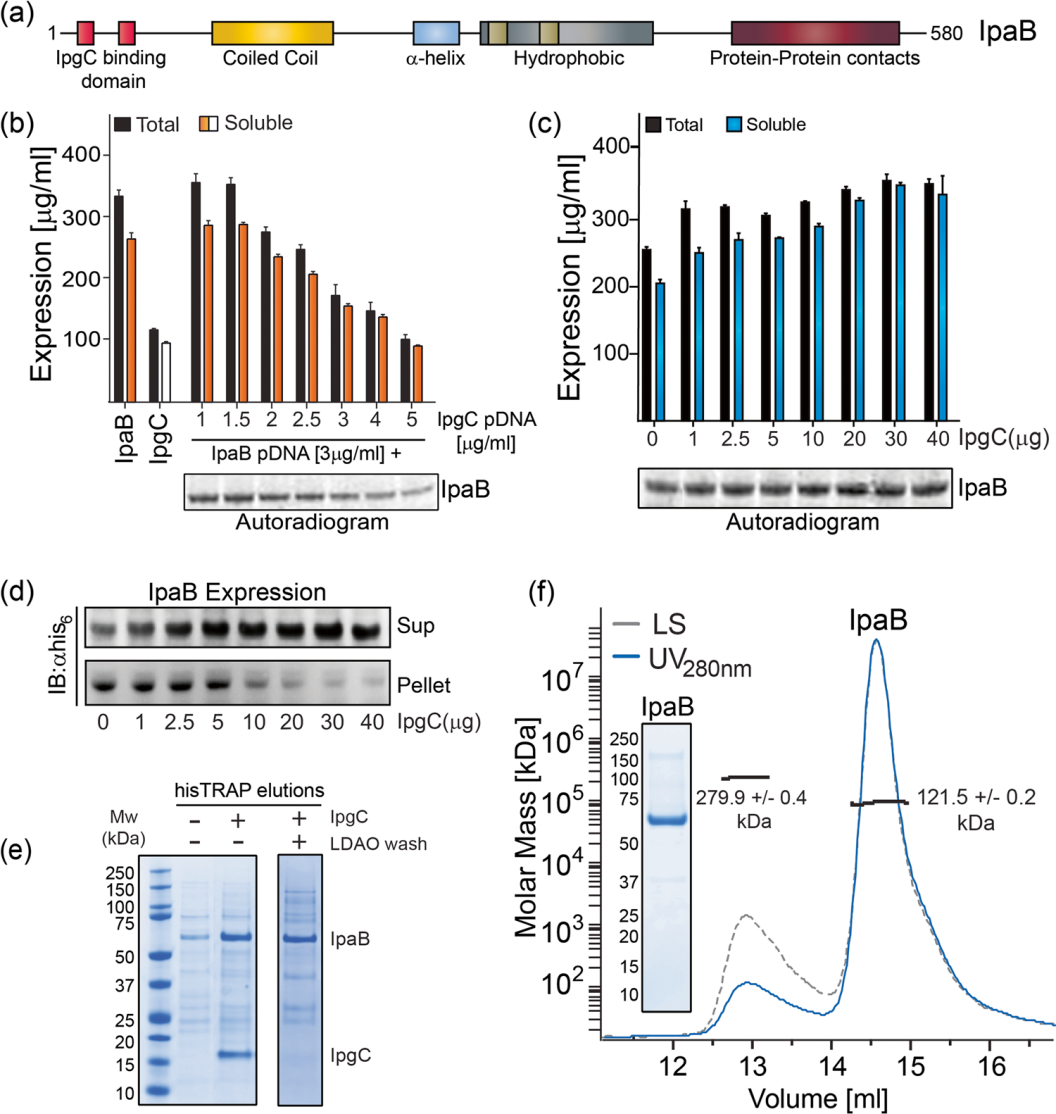
Expression, purification, and biophysical characterization of IpaB. **a** Modular architecture of IpaB indicating IpgC binding domains near the N-terminus. IpaB co-expressed with IpgC in CFPS reaction containing increasing amounts of IpgC pDNA (**b**) and IpaB expressed alone when increasing amounts of purified IpgC were added to the CFPS reaction (c); protein amount was determined using ^14^C-leucine incorporation and data represent mean protein concentration ± SD from 3 independent measurements. **d** Western blot analysis of the supernatant and pellet fractions from co-expression of IpaB with increasing amounts of purified IpgC in scaled up thin-layer reactions shows complete recovery of IpaB into the soluble fraction when > 30 μg of IpgC was added. **e** SDS-PAGE analysis shows significantly higher amounts of IpaB recovered in the elution fractions when purified IpgC was exogenously added into the reaction mixture (**f**) SEC-MALS analysis of purified IpaB (inset, > 95% purity as assessed by SDS-PAGE analysis) shows primarily dimeric state of the protein in solution.

Structurally, *Shigella* IpaB has a modular architecture that includes the cytosolic chaperone IpgC binding domain (aa 15-72) (Ferrari et al. 2021; Lokareddy et al. 2010), a coiled-coil region (aa 74-239 or 110-170 or 85-200) (Ferrari et al. 2021; Oaks and Turbyfill 1992), a central hydrophobic domain, and a putative protein binding region towards the C-terminal end of the protein (Fig. 3a) (Guichon et al. 2001; Shen et al. 2010). IpaB has traditionally been purified using cell-based expression platforms (i.e., *E. coli*) (Barta et al. 2017; Hume et al. 2003; Martinez-Becerra et al. 2012). This method was used to produce modest quantities of IpaB with acceptable purity for biophysical and functional analyses (Barta et al. 2018), as well as preclinical studies on this potential vaccine candidate (Chitradevi et al. 2016; Heine et al. 2013; Martinez-Becerra et al. 2012). We have adapted and optimized the cell-free Xpress^+TM^ CFPS to produce large quantities of pure, soluble IpaB for immunological and vaccine studies. Expression of IpaB alone at room temperature resulted in > 200 mg/L of soluble protein, as estimated through ^14^C-leucine incorporation into the translating polypeptide (Fig. 3b). However, SEC-MALS analysis revealed aggregated protein (data not shown). IpaB is kept in its native non-aggregated form in the bacterial cytosol through interaction with its cognate molecular chaperone, IpgC (Ménard et al. 1994; Picking et al. 1996; Picking and Picking 2016). IpaB binding to IpgC also prevents degradation; thus, co-expression of IpaB with IpgC in *E. coli* enabled viable purification of IpaB (Birket et al. 2007; Lokareddy et al. 2010). Building on these observations, we utilized the open nature of the CFPS to co-express full-length IpaB while adding to the reaction mix increasing amounts of IpgC-encoding plasmid DNA (pDNA) using a 96-well plate reaction format. The incremental addition of *ipg*C expression pDNA dramatically lowered the expression of IpaB (Fig. 3b), possibly due to dominant expression of IpgC itself at higher pDNA concentrations. To circumvent this limitation, we examined IpaB expression with increasing amounts of purified IpgC protein (up to 40 μg, i.e., 28 μM) instead of *ipg*C expression pDNA added to the reaction mix. The addition of IpgC into the cell-free media improved both the total and soluble IpaB yields, reaching saturation at > 30 μg (~ 21 μM) of IpgC (Fig. 3c**)**. Using these conditions, we next investigated the effect of IpgC on IpaB aggregation and precipitation. Exogenous addition of purified IpgC (up to 30 μg) eliminated IpaB precipitation and allowed for complete solubilization of IpaB, as shown by Western Blot analysis (Fig. 3d**)**. Finally, we scaled up expression of IpaB to 1L alone or with IpgC at 25 °C and pH 7.2 using a DASbox bioreactor. His_6_-tagged IpaB was captured from the clarified reaction mix supernatant. SDS-PAGE analysis revealed higher amounts of IpaB in the elution fractions when purified IpgC was added to the CFPS reaction (Fig. 3e**).** Importantly, the co-eluting chaperone was effectively removed from IpaB, by washing the column bound protein fraction with loading buffer supplemented with the zwitterionic detergent lauryl diamine oxide (LDAO). Thereafter, the consolidated HisTrap elution fractions were subjected to size exclusion chromatography using a pre-equilibrated Superdex200 26/60 column and the elution fractions were analyzed by SDS-PAGE. Highest purity (> 95%) IpaB-containing fractions were combined, concentrated, aliquoted, and stored at – 80 °C. Finally, SEC-MALS analysis performed on purified IpaB showed that the CFPS-produced IpaB had a molecular mass of 121.5 ± 0.2 kDa, which is a close approximation to the theoretical mass of 124.2 kDa for an IpaB dimer.

### Expression, purification, and biophysical characterization of cell-free generated IpaH-CTD

**Fig. 4:**
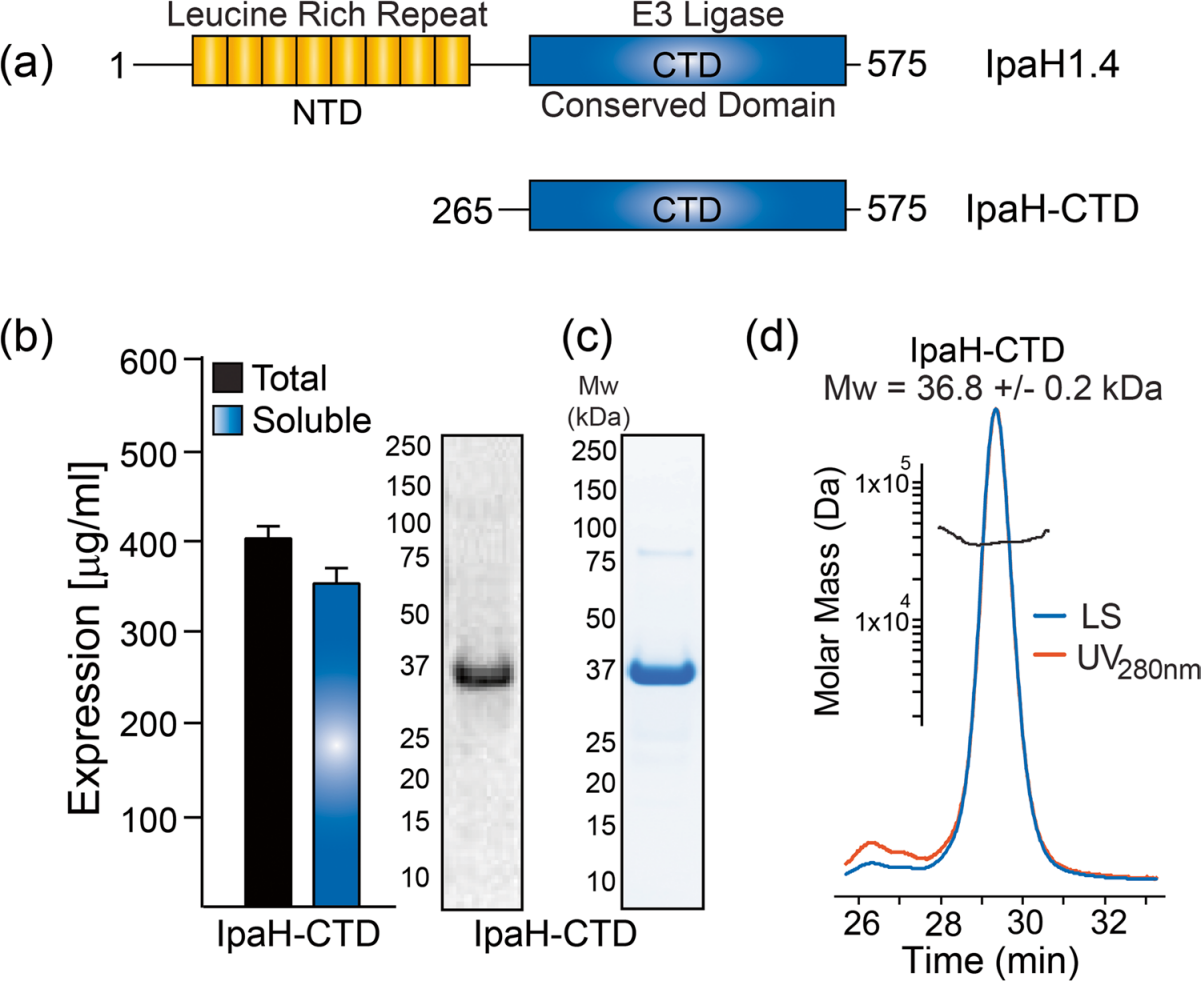
Expression, purification, and characterization of IpaH-CTD generated using CFPS platform. **a** Schematic representation of full length IpaH1.4 illustrating the leucine repeat–rich N-terminal (NTD) protein-protein interaction domain followed by the highly conserved C-terminal catalytic domain (CTD) with E3 ligase activity. **b** Using ^14^C-leucine incorporation, expression yield of IpaH-CTD was estimated > 300 μg/ml with a single band corresponding to IpaH-CTD in the autoradiogram. Bars represent mean protein concentration ± SD from 3 independent measurements. **c** SDS-PAGE analysis of the purified protein followed by SimplyBlue^TM^ SafeStain staining shows a single band with purity > 95%. **d** SEC-MALS analysis of purified IpaH-CTD shows monomeric state in solution

Similar to IpaB, the IpaH family of virulence proteins (encoded on both the chromosome and virulence plasmid) are secreted through the T3SS. Structurally, all members harbor an N-terminal leucine-rich repeat region domain (NTD) followed by a C-terminal catalytic core domain (CTD) that possesses E3 ligase activity (Ashida et al. 2007) (Fig. 4a). IpaH has been primarily a gene/antigen used for diagnostic purposes (Lindsay et al. 2013; Liu et al. 2016; Sahl et al. 2015). While the N-terminal domain varies among the 9 IpaH proteins, the C-terminal domain is conserved between all isoforms (Ashida and Sasakawa 2015). As described for IpaB, we adapted and optimized the CFPS platform to express and purify large amounts of the IpaH1.4 C-terminal domain (IpaH-CTD). IpaH1.4 was one of the top isoforms recognized by serum from vaccinated or *S.flexneri 2a*–challenged individuals using a core *Shigella* proteome microarray (Ndungo et al. 2018). Using ^14^C-leucine incorporation, the total and soluble IpaH-CTD was estimated to be > 300 mg/L (Fig. 4b). SDS-PAGE analysis of the CFPS reactions revealed a single protein fragment in the autoradiogram (Fig. 4b). Thereafter, expression of N-terminally his_6_-tagged IpaH-CTD was scaled up at 25 °C and pH 7.2 in a DASbox bioreactor. Post-expression his_6_-tagged IpaH-CTD was captured from the clarified supernatant using HisTrap column chromatography. Thereafter, the elution fractions were combined, concentrated, and subjected to size exclusion chromatography using a Superdex 75 16/60 column. The elution fractions were analyzed by SDS-PAGE followed by safe-blue staining (Fig. 4c). The highest purity fractions (> 95%) were combined and concentrated, aliquoted, and stored at – 80 °C. The recovery yields of IpaH-CTD were estimated to be ~ 200 mg/L using A_280_ absorbance. The in-solution biophysical state of the purified IpaH-CTD (Fig. 4d) was investigated by SEC-MALS analysis, which estimated a molecular mass of 36.8 ± 0.2 kDa; this value is in close agreement with the theoretical molecular mass of 36.4 kDa for an IpaH-CTD monomer.

### Immune reactivity of *Shigella* IpaB and IpaH-CTD produced in the CFPS

**Fig. 5:**
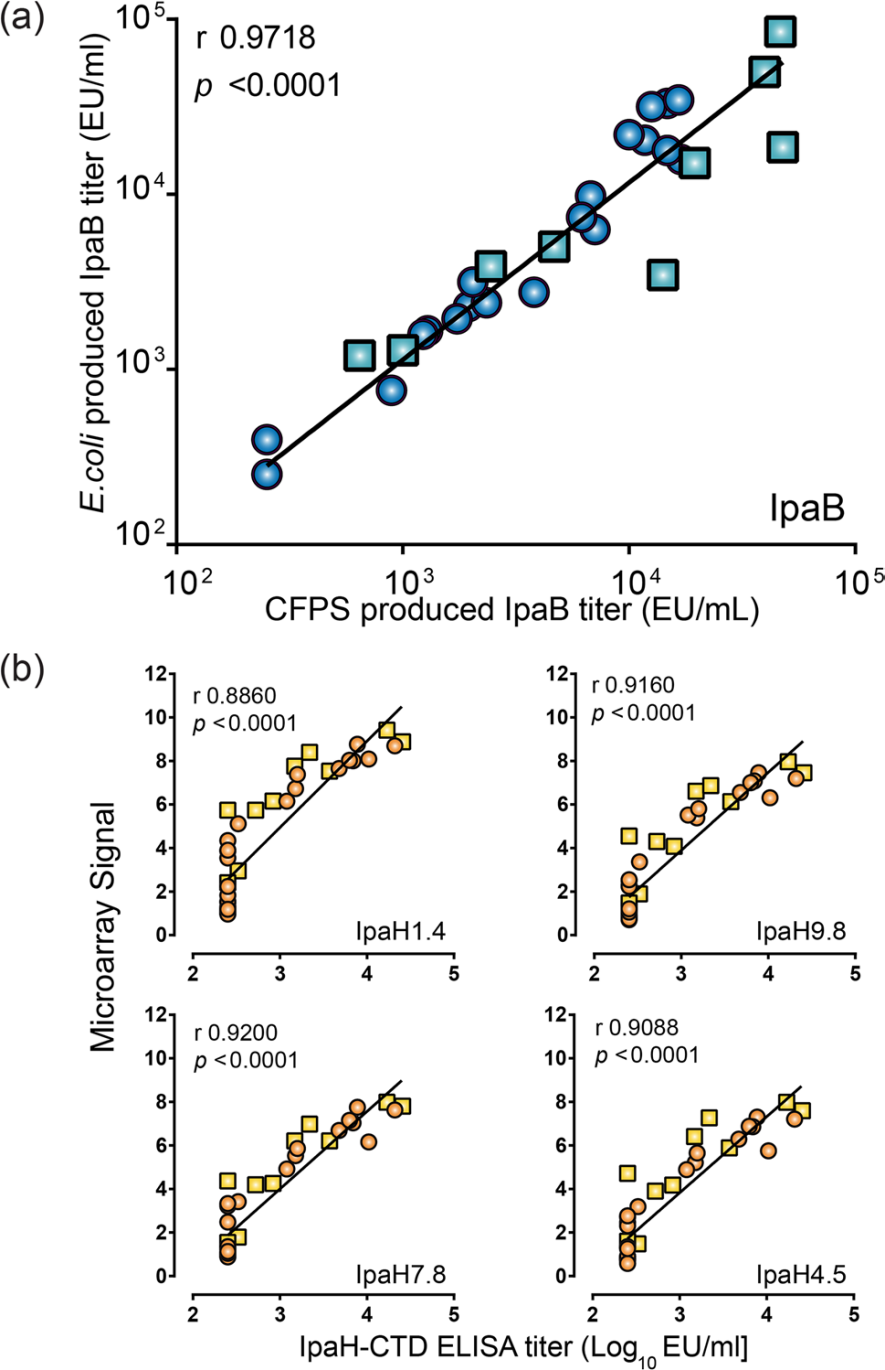
Immune reactivity of IpaB and IpaH-CTD produced in CFPS. Serum IgG titers against IpaB (**a**) or IpaH-CTD (**b**) in 14 volunteers pre- and post-challenge with *S.flexneri 2a* (circles) and 5 volunteers pre- and post-immunization with live-attenuated *S.flexneri 2a* (squares) measured by ELISA using IpaB or IpaH-CTD produced in CFPS versus titers obtained by ELISA using IpaB produced in *E. coli* (**a**) or versus IpaH-specific antibody binding signals obtained in a protein microarray (**b**). Pearson correlation coefficient (r) and *p* value are indicated

To determine the immune reactivity of CFPS-purified IpaB and IpaH-CTD, we evaluated their recognition by serum antibodies from individuals challenged with WT *S.flexneri 2a* (Kotloff et al. 1995) or orally immunized with a live-attenuated *S.flexneri 2a* vaccine (CVD 1204) (Kotloff et al. 2004). IpaB antibody binding was determined by ELISA using IpaB produced in *E. coli*; this method had been used by our group and others (Frenck Jr. et al. 2018; Shimanovich et al. 2017) . Serum antibody titers measured against IpaB produced in *E. coli* and CFPS were compared and found to be almost identical in absolute numbers and strongly correlated (Fig. 5a). These results demonstrate that immune reactive epitopes are maintained in the CFPS-purified IpaB and that the absolute antibody content detected was similar regardless of the purification method. Immune responses to IpaH have not been previously evaluated in *Shigella* clinical studies as sufficient purified (and well-characterized) IpaH was not available. To evaluate immunoreactivity of CFPS IpaH-CTD, antibody titers obtained using this protein as coating antigen in an ELISA (similar to that described above for IpaB) were compared to normalized antibody binding signals against full-length IpaH1.4 that were obtained using the microarray platform previously described (Ndungo et al. 2018). A strong correlation was found between the ELISA titers and the microarray signal intensities as shown in Fig. 5b. Interestingly, IpaH-CTD ELISA titers were also correlated with the normalized signal intensities to three other IpaH isoforms on the microarray (Fig. 5b). These results confirm the antigenicity of IpaH-CTD and show that antibodies directed to the conserved C-terminal domain can target multiple IpaH isoforms. IpaH produced in *E. coli* was not available for side-by-side antibody titer comparison.

### Immunogenicity of CFPS-produced IpaB and IpaH-CTD in mice

**Fig. 6:**
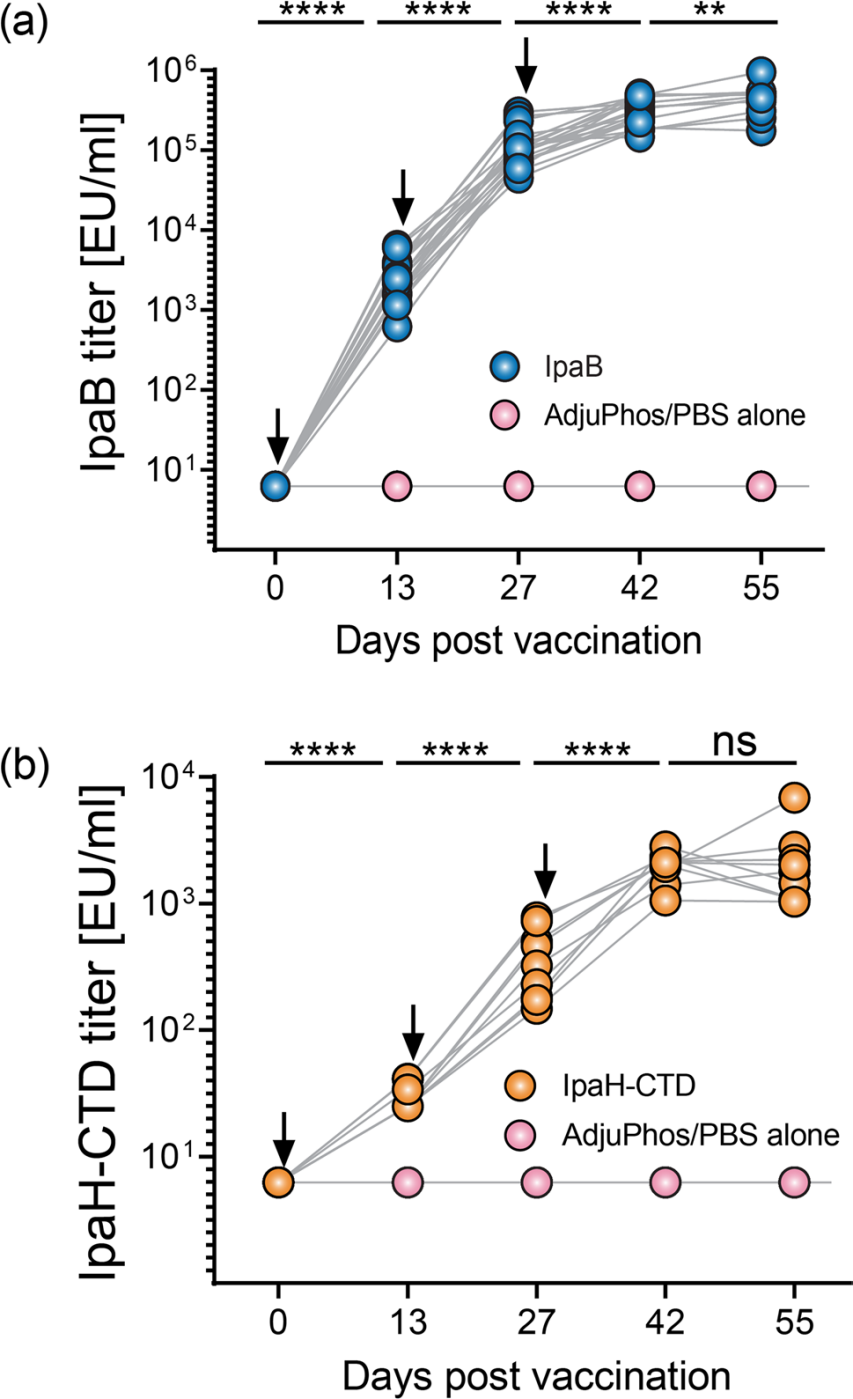
Immunogenicity of *Shigella* IpaB and IpaH produced in the CFPS. Adult female BALB/c mice (10–20/group) were immunized intramuscularly on days 0, 14, and 28 (arrows) with 10 μg of CFPS-produced IpaB (**a**) and IpaH-CTD (**b**) adjuvanted with Adju-Phos. Serum IgG titers were measured on days -1, 13, 27, 42, and 55. Data represent titers in each individual animal. Titers over time were compared by paired-*t* test analysis. Not significant (ns), *p* < 0.005 (**), *p*< 0.0001 (****)

We had previously shown that IpaB is a strong immunogen in mice immunized via the oral, intranasal, and intradermal routes (Heine et al. 2014; Heine et al. 2013; Martinez-Becerra et al. 2012). Here, we further demonstrated that IpaB and IpaH-CTD admixed with Adju-Phos® and administered intramuscularly to adult BALB/c mice were well tolerated and generated strong serum antibody responses (Fig. 6a and b). Antibody titers to both proteins improved after each subsequent vaccination. IpaB-specific titers continued to increase even 4 weeks after the third vaccine dose (the last time point measured) while antibody titers specific to IpaH-CTD remain unchanged between days 42 and 55 (Fig. 6a and b). These results confirm the in vivo immunogenicity of both proteins and the immunodominant properties of IpaB.

## Discussion

A safe, effective, simple to manufacture, and affordable *Shigella* vaccine that can reduce moderate to severe diarrhea in young children in low- and middle-income countries can have a major public health impact. Travelers and military personnel deployed to endemic regions could also benefit from such a vaccine. Clinically advanced vaccine candidates rely on generating immunity against the bacterial O-antigen (Cohen et al. 2021; Riddle et al. 2011; Walker et al. 2021). A major limitation of this approach is restricted coverage and at least a quadrivalent formulation would be needed to afford immunity against 60–70% of the most prevalent circulating *Shigella* strains (Livio et al. 2014; Noriega et al. 1999). The need for a multivalent vaccine also complicates clinical evaluation and increases cost of vaccine manufacturing. Most importantly, the effectiveness of such a vaccine in young children, who failed to develop protective levels of *Shigella* LPS antibodies in response to an early administration of a protein-conjugate vaccine (Passwell et al. 2010), remains to be demonstrated.

Similar to other enteric pathogens, *Shigella* has a T3SS apparatus that enables translocation of virulence factors required for bacterial infection of the human colonic epithelium. T3SS proteins, primarily IpaB, IpaC, and IpaD, are known to stimulate a robust host immune response after infection and have been identified as potential *Shigella* vaccine targets (Chitradevi et al. 2016; Martinez-Becerra et al. 2012; Turbyfill et al. 2018). Because the Ipa proteins are highly conserved among *Shigella* species, a vaccine based on Ipa proteins would afford broad coverage. A protein-only vaccine would be easier to consistently manufacture and more economical than an O-antigen based multicomponent vaccine. There is precedent of successful routine immunization of children with parenterally delivered protein-based vaccines to protect communities (Anderson et al. 2018; Briere et al. 2014; Guris et al. 1997; Turbyfill et al. 2018). The clinical advancement of a *Shigella* protein vaccine would require an efficient and scalable production method in place. The CFPS system described herein represents a novel GMP-compatible platform technology for large-scale production of IpaB and IpaH-CTD that overcomes the yield limitations of cell-based methods.

IpaB is highly immunogenic and a putative protective antigen. IpaB-specific serum IgG has been associated with clinical protection in experimentally challenged individuals, (Shimanovich et al. 2017), and multiple studies have reported the protective capacity of IpaB in mice (Chitradevi et al. 2016; Heine et al. 2014; Heine et al. 2013; Heine et al. 2015; Martinez-Becerra et al. 2012). Yet, IpaB has never been tested as a vaccine candidate in humans. The evaluation of vaccines in clinical studies requires large quantities of a highly pure and well-characterized investigational product with an acceptable stability profile. Similarly, high-quality antigens are needed for immunological analysis. Research-grade IpaB produced in *E. coli* used for research purposes did not meet requirements for clinical evaluation. An attempt to produce a *Shigella* IpaB-D fusion (Martinez-Becerra et al. 2013a) for a human phase 1 study failed at the manufacturing stage, and the clinical development program (supported by PATH) was halted (Wilbur Chen, personal communication). The CFPS platform reported here allowed for scaled-up production of highly pure and soluble IpaB. The CFPS-IpaB performed similarly to *E. coli*–derived IpaB when used as coating antigen in a traditional indirect ELISA. The CFPS-IpaB administered to mice intramuscularly adjuvanted with alum was immunogenic and elicited a robust serum IgG response, even after a single vaccination. Thus, our results indicate that not only is IpaB produced by CFPS comparable to *E. coli*–produced IpaB, but it can also be efficiently produced at high yields (> 200 mg/L) greatly exceeding the low milligram IpaB yields reported in the literature using conventional cell-based methods (Picking et al. 1996).

The *ipa*H gene family has been successfully utilized as a molecular diagnostic marker, but unlike IpaB, the immunogenicity of IpaH has not been studied. We have reported that IpaH was recognized by circulating antibodies from orally vaccinated or *S.flexneri 2a*–challenged individuals (Ndungo et al. 2018). Herein, we showed that the CFPS generated IpaH-CTD was also similarly recognized by antibodies from *Shigella-*exposed subjects, which demonstrates that IpaH-CTD maintained immunoreactive epitopes. Antibody reactivity to the conserved C-terminal domain was associated with signals from multiple IpaH isoforms, confirming the conserved nature of the purified protein. In addition, purified IpaH-CTD elicited strong serum IgG immune responses in mice, confirming its immunogenicity. Availability of the IpaH-CTD in large quantities would allow investigations on its structure and its potential use as an immunodiagnostic tool and vaccine candidate. The conserved nature of the C-terminal domain suggests that the purified protein could be helpful in facilitating further studies of members of the IpaH family of proteins.

The CFPS platform is being implemented for production of additional Ipas (i.e., IpaA, IpaC, and IpaD). Access to large quantities of highly pure *Shigella* T3SS proteins offers the opportunity for biophysical and functional studies to better understand the mechanisms by which these proteins interact with other bacterial and host elements. High-resolution structural information and details on precise immunological priming and effector mechanisms of these proteins, particularly in relation to protective immunity, can help guide the design of safe and effective vaccines. The CFPS approach can also be extended to obtain T3SS proteins from other pathogens.

A variety of proteins from growth factors, toxins, and viral-like particles (Chiba et al. 2021; Gao et al. 2021; Zawada et al. 2011; Zichel et al. 2010) have been produced successfully using CFPS. In addition, complex antibody drug conjugates have been generated using the CFPS and linearly scaled from research to GMP manufacturing levels for evaluation in clinical trials (Abrahams et al. 2018). The open nature of the platform incentivizes real-time sampling and manipulation of the reaction conditions to optimize protein expression and solubility. Unlike cell-based systems, the absence of membranous architecture permits configurational flexibility by allowing addition of surfactants/lipids and biochemical components to influence pH and oxidative potential and improve solubility of protein targets that would otherwise be hard to generate (Jewett and Swartz 2004; Kapoor et al. 2018; Kim and Swartz 2004). Furthermore, unlike cell-based systems, which require cloning and transformation of cells to initiate antigen production, CFPS reduces the process completion time from weeks to hours, as the synthesized gene once cloned into the expression vector can be immediately utilized to initiate protein expression. Importantly, the lack of post-translational modification machinery also promotes generation of molecularly consistent proteins that are easier to biophysically characterize at scale (Yin et al. 2012). Finally, initiation of protein purification does not require time-consuming steps like cell lysis, as the harvested cell-free reaction can just be centrifuged or depth filter clarified for immediate loading onto a capture column for protein purification. These major process improvements result in significant cost reductions for production of affordable vaccine targets (Chiba et al. 2021; Sheng et al. 2017; Zichel et al. 2010). Using CFPS, Sheng et al. (Sheng et al. 2017) estimated that generation time of norovirus viral-like particle vaccine was > 40h less, compared to cell-based systems, while the cost/dose was still within the $2.5–5.0/dose range of cell-based systems. Such improvements in process efficiencies can provide significant cost savings for GMP production of a *Shigella* vaccine, which will primarily be deployed (and most needed) in resource-poor regions across the world. In summary, CFPS is a scalable, simple, fast, practical, and robust GMP-compatible platform for efficient synthesis of biological products. The CFPS technology opens the way for efficient scale-up of production of well characterized *Shigella* vaccine targets like IpaB and IpaH for research studies and to support phase 1 clinical trials.

## Data Availability

The datasets generated and/or analyzed during the current study are available from the corresponding authors on reasonable request.
